# P-647. Comparative efficacy of antibiotic regimens against *Ureaplasma* species with varying antibiotic resistance profiles in a mouse model of lung infection

**DOI:** 10.1093/ofid/ofae631.844

**Published:** 2025-01-29

**Authors:** Derek Fleming, Robin Patel

**Affiliations:** Mayo Clinic, Rochester, Minnesota; Mayo Clinic, Rochester, Minnesota

## Abstract

**Background:**

Lung transplant recipients (LTRs) can experience hyperammonemia syndrome (HS) correlated with lung infection by ammonia-producing *Ureaplasma* species. HS in LTRs can be managed by *Ureaplasma*-directed antibiotic therapy; however, *Ureaplasma* species lack a cell wall, limiting treatment options. Precise guidelines for *Ureaplasma*-directed antibiotic therapy in LTRs is lacking, and antibiotic resistance may complicate treatment. Here, levofloxacin (LEV), azithromycin (AZM), and doxycycline (DOX) were tested against isolates of *U. urealyticum* and *U. parvum* with varying antibiotic resistance profiles, and efficacies compared.

**Fig 1.**
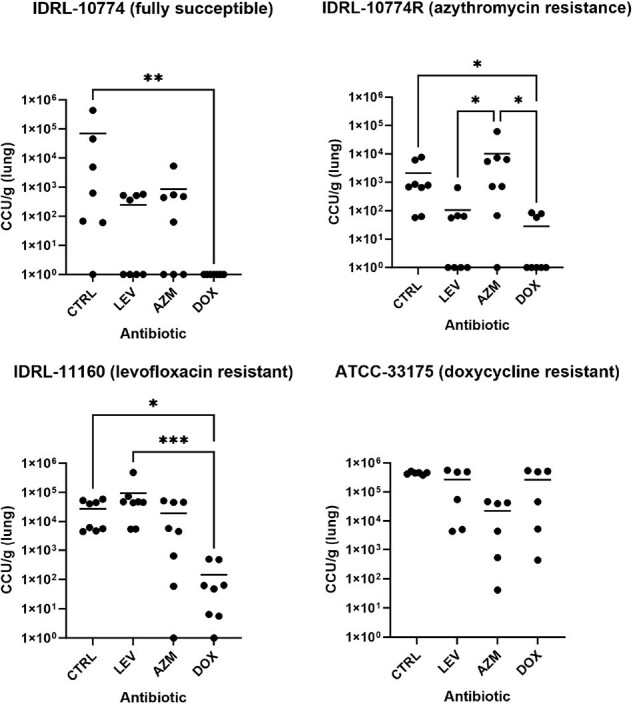
Antibiotic prophylaxis of murine infection by antibiotic resistant Ureaplasma isolates.

Mice were prophylaxed with levofloxacin (LEV; 100 mg/kg SQ), azithromycin (AZM; 100 mg/kg PO), or doxycycline (DOX; 100 mg/kg IP) 12 hours prior to infection with isolates of Ureaplasma parvum or Ureaplasma urealyticum resistant to one or none of the antibiotics. 18 hours after infection, mice were sacrificed and lungs harvested, with resulting Ureaplasma loads quantified (color changing units: CCU). Control animals (CTRL) were given no antibiotics. Significance between groups was determined via Kruskal-Wallis tests with Dunn’s multiple comparison tests. * p ≤0.05, **p ≤0.01, ***p ≤0.001.

**Methods:**

Mice were immunosuppressed using a combination of tacrolimus (1.2 mg/kg daily), mycophenolate mofetil (40 mg/kg daily), and methylprednisolone (20 mg/kg weekly) to mimic immunosuppression given to LTRs. Prophylactic antibiotics were given 12 hours prior to infection, including either LEV (100 mg/kg subcutaneously), AZM (100 mg/kg orally), or DOX (100 mg/kg intraperitoneally). Mice were then infected with *Ureaplasma* isolates resistant to one or none of the antibiotics, including *U. parvum* IDRL-10774 (fully susceptible), *U parvum* IDRL-10774R (IDLR-10774 with evolved AZM resistance; serial passage in sub-MIC AZM), *U. parvum* IDRL-11160 (LEV resistant), and *U. urealyticum* ATCC-33175 (DOX resistant). Mice were euthanized 18 hours post-infection, and lung bacterial loads (color changing units; CCU/gram) quantified.

**Results:**

The effect of various antibiotic treatments against mouse lung infections with the four *Ureaplasma* isolates is displayed in **Fig 1**. Infection with IDRL-10774 (fully susceptible) and IDRL-11160 (LEV resistant) was only prevented by doxycycline (p=0.09 and p=0.01, respectively). For IDRL-10774, insignificant mean reductions (possibly due to a high standard deviation in the controls) were observed for both LEV (-10^6.9^ CCU/g) and AZM (-10^5.25^ CCU/g). IDRL-10774R (evolved AZM resistance) showed a complete loss of susceptibility to AZM compared to unevolved IDRL-10774. ATCC-33175 (DOX resistant) was not reduced by any antibiotics tested.

**Conclusion:**

DOX was the most effective antibiotic tested, preventing lung infection by all isolates, except a DOX resistant isolate.

**Disclosures:**

**Robin Patel, MD**, a patent on Bordetella pertussis/parapertussis PCR issued, a patent on a device/method for sonication with royalties paid by Samsung to Mayo Clinic, a: See above|MicuRx Pharmaceuticals and BIOFIRE: Grant/Research Support|PhAST, Day Zero Diagnostics, Abbott Laboratories, Sysmex, DEEPULL DIAGNOSTICS, S.L., Netflix, Oxford Nanopore Technologies and CARB-X: Advisor/Consultant|Up-to-Date and the Infectious Diseases Board Review Course.: Honoraria

